# P04-01 Slovenian approach to healthy and active aging - Fall prevention program for elderly living at home

**DOI:** 10.1093/eurpub/ckac095.055

**Published:** 2022-08-29

**Authors:** Tjaša Knific, Martina Horvat, Andrea Backović Juričan

**Affiliations:** Prevention and Promotion Programmes Management, National Institut of Public Health, Ljubljana, Slovenia

**Keywords:** fall prevention, elderly, active aging

## Abstract

**Issue/problem:**

Due to the population aging, injuries in relation to falls are a public health problem in Slovenia as well. They are expensive for the health care system, usually have serious consequences and lead to irreversible impairment of function, institutionalization and death.

Description of the problem

During 2019, registered nurses in community health nursing from 25 Primary Healthcare Centres participated in the MoST pilot project. They conducted a fall risk assessment of 64 years and older patients at their homes, using a Slovenian adaptation of the STEADI (Stopping Elderly Accidents, Deaths & Injuries) questionnaire. At the same time, they also performed an assessment of muscle strength, gait and balance with a timed framed Stand up and go test. Afterwards patients were classified into 3 groups (low, medium, and high risk of falls) and were treated according to the Algorithm for the assessment of the risk of falls for the elderly and prevention measures.

**Results (effects/changes):**

6815 fall risk assessments were conducted (results are preliminary). More details will be presented at the HEPA conference as the data is still being processed. Based on the expected population distribution, mobility and nutrition treatment, home safety counselling and a multifactorial risk assessment during the development phase of the project were planned. The interventions were performed at integrated Health Promotion Centres in Primary Healthcare Centres and at the homes of the elderly.

**Lessons:**

The risk assessment method described earlier should be carried out at the home of an elderly person. The most effective interventions were multicomponent measures or prevention programs with ever-present physical exercise program. Focused physical activity aiming at improving strength and balance not only reduces the frequency of falls, but also the frequency and severity of injuries in the event of a fall.

**Main messages:**

A fall risk assessment is an economically viable fall prevention intervention in the elderly. Time invested in preventing falls is reflected in a more active elderly population.

